# Identification of a putative germ plasm in the amphipod *Parhyale hawaiensis*

**DOI:** 10.1186/2041-9139-4-34

**Published:** 2013-12-05

**Authors:** Tripti Gupta, Cassandra G Extavour

**Affiliations:** 1 Department of Organismic and Evolutionary Biology, Harvard University, 16 Divinity Avenue, Cambridge, MA 02138, USA

**Keywords:** Germ plasm, Germ line, Arthropod, Amphipod, *vasa*, *orb*, *germ cell-less*, Cytoplasmic determinant

## Abstract

**Background:**

Specification of the germ line is an essential event during the embryonic development of sexually reproducing animals, as germ line cells are uniquely capable of giving rise to the next generation. Animal germ cells arise through either inheritance of a specialized, maternally supplied cytoplasm called 'germ plasm’ or though inductive signaling by somatic cells. Our understanding of germ cell determination is based largely on a small number of model organisms. To better understand the evolution of germ cell specification, we are investigating this process in the amphipod crustacean *Parhyale hawaiensis*. Experimental evidence from previous studies demonstrated that *Parhyale* germ cells are specified through inheritance of a maternally supplied cytoplasmic determinant; however, this determinant has not been identified.

**Results:**

Here we show that the one-cell stage *Parhyale* embryo has a distinct cytoplasmic region that can be identified by morphology as well as the localization of germ line-associated RNAs. Removal of this cytoplasmic region results in a loss of embryonic germ cells, supporting the hypothesis that it is required for specification of the germ line. Surprisingly, we found that removal of this distinct cytoplasm also results in aberrant somatic cell behaviors, as embryos fail to gastrulate.

**Conclusions:**

*Parhyale hawaiensis* embryos have a specialized cytoplasm that is required for specification of the germ line. Our data provide the first functional evidence of a putative germ plasm in a crustacean and provide the basis for comparative functional analysis of germ plasm formation within non-insect arthropods.

## Background

A key event during the development of sexually reproducing organisms is the formation of germ cells, the cells that ultimately give rise to the next generation. In all sexually reproducing animals that have been examined, germ cells are specified by inheritance of a specialized cytoplasm called 'germ plasm’ or though zygotic induction by somatic cells. Although the inheritance of germ plasm is widespread amongst metazoans, only three phyla (rotifers, nematodes and chaetognaths) appear to be entirely characterized by this mechanism of germ cell specification, and it is hypothesized that germ plasm evolved independently many times during evolution [1]. Interestingly, despite the evolutionary novelty of germ plasm within different phyla, germ plasm shares many conserved features amongst species [2-6]. Germ plasm is characteristically yolk-free and non-membrane bound, with a concentration of mitochondria and maternally deposited RNAs that are often associated with organelles called germinal granules [6,7]. In all species examined, germ plasm contains products of the *vasa* gene, a conserved ATP-dependent helicase [8,9].

The first functional tests of germ plasm were performed on chrysomelid beetle embryos by Hegner in 1908 when he removed the posteriorly localized germ plasm and observed that the embryos developed without germ cells [10,11]. In 1974, Illmensee and Mahowald performed seminal experiments using *Drosophila* and transplanted germ plasm from the posterior pole to the anterior and lateral regions of the embryo to produce ectopic germ cells, thereby demonstrating the sufficiency of germ plasm for germ line specification [12]. A recent study in which ectopic germ cells were induced by transplantation of germ plasm in *Xenopus* demonstrated that germ plasm is sufficient to determine germ cells in a vertebrate [13]. Thus, it has been shown that for some members of both vertebrate and invertebrate phyla, inheritance of germ plasm is both necessary and sufficient for germ line specification.

The Arthropoda is the largest and most diverse animal phylum; however, our knowledge of the mechanisms of arthropod germ plasm formation derive almost exclusively from a single species, *Drosophila melanogaster*. In *Drosophila*, germ cell formation is dependent on the germ plasm nucleator Oskar, a novel protein that is only found in insects [14,15]. In addition, *Drosophila* germ cells form as pole cells at the posterior of the embryo, a feature unique to holometabolous insects [16] (but see [17] for data from a thrips). Identification of primordial germ cells in crustaceans has been based almost exclusively on cytological analysis and expression of the germ line marker *vasa*. Although evidence exists by these criteria for the presence of germ plasm in several species (for example [18,19]), functional analyses to test the necessity and sufficiency of a cytoplasmic determinant for germ cell specification have not been performed in a crustacean.

To gain a broader understanding of the mechanisms of germ line development in arthropods, we are using the amphipod crustacean *Parhyale hawaiensis* to study the process of germ cell specification. *Parhyale* embryos undergo holoblastic cleavages, and in wild type embryos cell fate is thought to be determined by cell lineage [20,21]. The first three cleavages in *Parhyale* result in an embryo with four macromeres and four micromeres. The four macromeres give rise to the anterior and visceral mesoderm (Mav), the right anterior ectoderm (Er), the left anterior ectoderm (El) and the posterior ectoderm (Ep). The micromeres give rise to the germ line (g), the right trunk mesoderm (mr), the left trunk mesoderm (ml) and the endoderm (en) [20]. Although some somatic cell fates are capable of regulative replacement at late stages of embryogenesis [22], blastomere isolation and ablation experiments have shown that, consistent with the results of lineage tracing studies [20,23], early cell fates appear to be determined autonomously at least through gastrulation [21,23,24]. This early restriction of fates suggests that the earliest cleavages are polarized and may effect asymmetric distribution of maternal cell fate determinants.

Germ cells can be distinguished by the presence of high levels of *vasa* transcript and protein [21,25]. Lineage tracing as well as blastomere isolation experiments have demonstrated that the g micromere is the exclusive source of germ line cells in *Parhyale*[20,21]. Blastomere isolation experiments, in which individual blastomeres of the eight-cell embryo were isolated, cultured and assayed for Vasa expression, also provided evidence for the existence of an inherited cytoplasmic determinant, but did not specifically identify this determinant [21].

In this study, we report the presence of a distinct cytoplasmic region in the one-cell *Parhyale* embryo that is required for specification of the germ line. We show that this cytoplasmic region contains conserved germ line transcripts, and that when this region is removed, the embryo develops without germ cells, consistent with a function as germ plasm. Surprisingly, this specialized cytoplasm also appears to be required for additional aspects of somatic cell fate, as embryos in which the cytoplasm has been removed appear to have defects in polarity and die prior to gastrulation. This work provides the first functional analysis of a putative germ plasm in a crustacean.

## Methods

### Animal culture

*Parhyale hawaiensis* adults were cultured in artificial seawater (Instant Ocean) with crushed coral at 28°C. Animals were fed daily with ground aquaculture feed (40% TetraPond® wheat germ sticks, 40% TetraMin® flake food and 20% Tropical® spirulina). Gravid females were anesthetized with CO_2_ and embryos were collected as previously described [26].

### Tissue fixation and staining

Embryos were fixed by incubation in 3.7% formaldehyde in 1× PBS for 2 minutes at 75°C followed by 20 minutes in 3.7% formaldehyde at 4°C. Membranes were then dissected from embryos in PBS and the embryos were fixed overnight at 4°C in 3.7% formaldehyde in 1× PBS. One-cell embryos were fixed when the RNA-containing body was clearly visible using incident illumination. Some embryos were stained with 10 nM SYTOX Green (Invitrogen).

### In situ hybridization

Whole mount *in situ* hybridization on *Parhyale* embryos was performed as described in [27] with the following modifications. Prior to rehydration, the embryos were cleared by incubation in xylene for 20 minutes. Following post-fixation, the embryos were incubated in detergent solution (1.0% sodium dodecyl sulfate (SDS), 0.5% Tween, 50.0 mM Tris–HCl (pH 7.5), 1.0 mM Ethylenediaminetetraacetic acid (EDTA; pH 8.0), 150.0 mM NaCl) for 30 minutes and then fixed again in 3.7% formaldehyde for 30 minutes. Hybridization was performed at 66°C or 67°C. After hybridization, the embryos were washed twice in 2× saline sodium citrate (SSC) for 30 minutes and then twice in 0.2× SSC for 30 minutes. Probes were visualized using NBT/BCIP (Sigma). Embryos were stained with 5 μg/ml Hoechst 33342 (Sigma). Sense probe controls showed no labeling (data not shown). Images were captured with an AxioCam MRm camera using AxioVision (Zeiss) and processed using Adobe Photoshop, Helicon Focus and ImageJ.

### Cloning and probe synthesis

Total RNA was isolated from the ovaries of adult *Parhyale* females using Trizol (Invitrogen) and used for first-strand cDNA synthesis with the SuperScript III 1st Strand Synthesis Kit (Invitrogen). *Parhyale orb* and *germ cell-less* orthologs were identified using sequences deposited in ASGARD [28] and primers for RT-PCR were designed using Primer3. cDNA fragments were cloned into the pCR4-TOPO vector (Invitrogen). Sense and anti-sense digoxygenin (DIG) labeled probes were transcribed using T3 and T7 polymerases.

Primers used for cDNA amplification were as follows:

*orb*: TGCTGAGCGCTTGTAATCAG

ATTGGCACCCAACTTTGAAC

*gcl*: CGAAAGCAACAGCACTTACG

CATCAGGGCTTCTTCCAGAC

Primers for cloning a 2505-bp fragment of *vasa* cDNA were based on a published sequence (Accession no. EU289291 [25]). Sense and anti-sense *vasa* probes were transcribed using T3 and T7 polymerases.

### Ablation of the RNA-containing body

The RNA-containing body (RCB) was removed from one-cell embryos approximately 2 hours after fertilization when it was clearly visible with incident illumination. To remove the RCB, an embryo was placed in a drop of filtered sterile seawater on a Sylgard silicone plate. The embryo was held with forceps and pierced with a glass needle in the center of the RCB. The embryo was then gently squeezed and the RCB and surrounding cytoplasm were extruded from the embryo. Control embryos were pierced with a glass needle at a random position approximately 100 μm away from the RCB along the long axis of the egg, and the embryos were gently squeezed so that the amount of non-RCB cytoplasm extruded from the control embryos was comparable to the amount of RCB cytoplasm removed from the experimental embryos. The embryos were then transferred to a dish of sterile filtered artificial seawater containing 100 units/ml penicillin, 100 μg/ml streptomycin and 1 μg/ml amphotericin and allowed to develop at 28°C. Any embryos that had not started to divide within 6 hours of ablation were assumed to be unfertilized and were discarded. Of the remaining embryos, 100% developed to the germ disc stage.

### Live imaging

Live images were captured with an AxioCam MRm camera using AxioVision (Zeiss). The embryos were illuminated with incident cold light and maintained at a temperature of 25°C in sterile artificial seawater. Scoring of g blastomere identity was confirmed by blind scoring by colleagues. Supplementary movies were edited using ImageJ and QuickTime Pro.

## Results

### Germ line-associated transcripts localize to a distinct cortical cytoplasmic region

**Figure 1:**
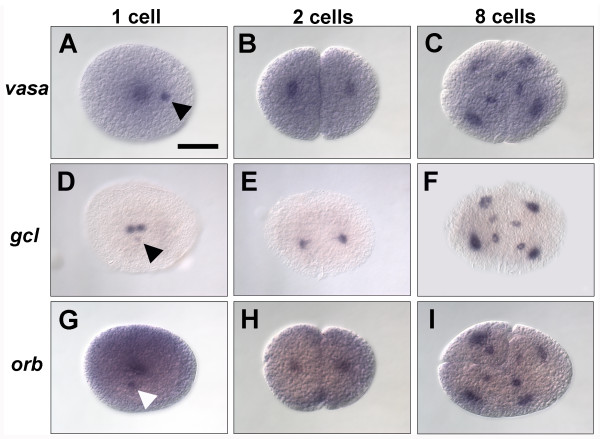
**Germ line-associated RNAs localize to a distinct cortical cytoplasmic region in one-cell embryos. (A–C)***vasa* transcript localization. **(A)** In one-cell embryos, *vasa* is localized to a distinct cytoplasmic region at the cortex of the embryo (arrowhead) and to the cytoplasm surrounding the nucleus. **(B)** In two-cell and **(C)** eight-cell embryos, *vasa* transcripts are predominantly localized to the cytoplasm surrounding the nuclei and are no longer seen in a distinct cortical domain. **(D–F)***gcl* transcript localization. **(D)** Like *vasa*, *gcl* is localized to a distinct cortical cytoplasmic domain in one-cell embryos (arrowhead). **(E,F)** In all stages subsequent to the first cell division, *gcl* transcripts are only detected surrounding the nuclei. **(G-I)***orb* transcript localization. **(G)** Similar to *vasa* and *gcl*, *orb* transcripts are localized to a distinct cytoplasmic region at the cortex of one-cell embryos (arrowhead) as well as to the cytoplasm surrounding the nucleus. **(H,I)** Following cell division, *orb* transcripts are predominantly localized to the cytoplasm surrounding the nuclei. Scale bar: 200 μm (all panels).

To characterize the expression of a set of germ line-associated genes, we examined their expression patterns by *in situ* hybridization in one-, two-, four- and eight-cell embryos. We found that transcripts of three such genes were localized to a distinct cortical cytoplasmic region in the one-cell embryo. The first gene, *vasa*, encodes an ATP-dependent RNA helicase and is a highly conserved germ line marker found in the germ plasm of all animals examined [1,8,29]. We examined the localization of *Parhyale vasa* mRNA by *in situ* hybridization and found that *vasa* transcripts are localized to a distinct cytoplasmic region at the cortex of one-cell embryos (Figure 1A, arrowhead; *n* = 17/17). In addition, as previously described, the transcript is localized around the nucleus of the embryo [25]. The transcript remains localized to the cortical cytoplasmic region as mitotic cell division begins, but by the two-cell stage, *vasa* transcripts can no longer be detected at the cortex and are only found surrounding the nuclei (Figure 1B). We also examined *vasa* expression in four- and eight-cell embryos, but consistent with previous observations [25], we only saw transcript localized around the cell nuclei and did not observe localization of *vasa* transcript to any other distinct cytoplasmic region in the g micromere or any of the other blastomeres (Figure 1C).

We next examined the expression of *germ cell-less* (*gcl*), a conserved BTB-domain (BTB: BR-C, ttk and bab) gene that localizes to germ plasm in *Drosophila*[30]. We found that *gcl* transcripts are also localized to a distinct cytoplasmic region at the cortex of one-cell *Parhyale* embryos (Figure 1D, arrowhead), and like *vasa*, the transcript can only be detected surrounding the nuclei after the first cell division (Figures 1E,F). Finally, we examined the expression of the *Parhyale* ortholog of *orb*, a cytoplasmic polyadenylation element binding protein that is localized to the germ plasm in *Drosophila*[31,32]. Like *vasa* and *gcl*, *orb* transcripts are localized to a cortical cytoplasmic region in one-cell embryos (Figure 1G, arrowhead), as well as around the nucleus, but after the first cell division, are only detected surrounding the nuclei of blastomeres (Figures 1H,I). *In situ* hybridization using sense probes for *vasa*, *gcl* and *orb* produced no signal, indicating that the cortical cytoplasmic region is not a non-specific attractor of labelled RNAs. In addition, we did not observe any staining in one-cell embryos using antisense probes for the zygotically expressed genes *cap-n-collar* and *spineless*, which label appendage primordia later in development (data not shown; P Sharma and TG, unpublished work) and play a role in somatic, but not germ line, development in other arthropods [33,34]. Taken together, these data indicate that there is a distinct cytoplasmic region in the one-cell embryo that is rich in germ line RNAs. Because of the accumulation of RNA localized to this region, we will hereafter refer to this distinct cytoplasmic region as the RNA-containing body (RCB).

### One-cell *Parhyale* embryos contain a distinct cytoplasmic region that corresponds to the RNA-containing body

**Figure 2:**
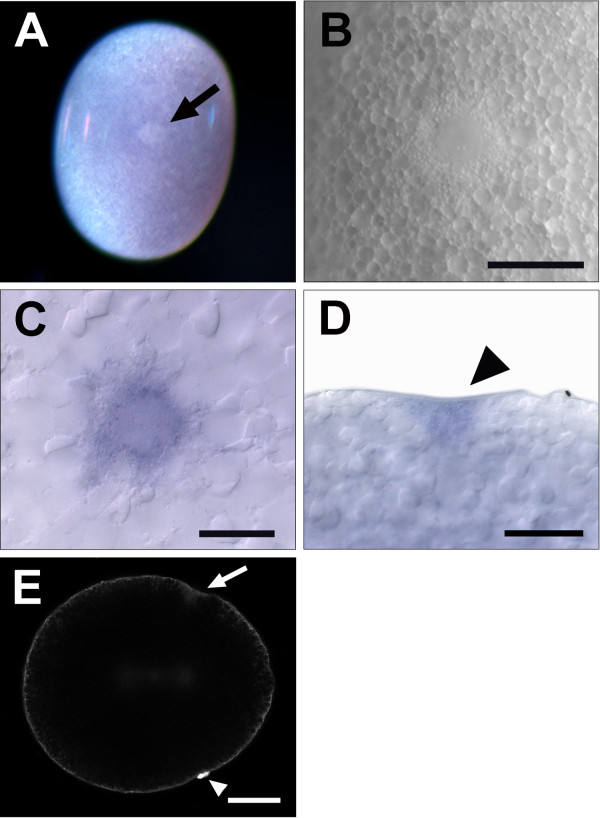
**One-cell *Parhyale *embryos have a distinct cortical cytoplasmic region that corresponds to the RCB. (A)** Following fertilization, a diffuse white spot that is more refractive than the surrounding cytoplasm appears at the cortex of the one-cell embryo (arrow). **(B)** The cortical cytoplasmic domain develops a ring-like appearance visible under incident illumination, with a yolk-free cytoplasmic area surrounded by small granules. **(C)***vasa* transcripts are localized to the RCB, which corresponds to the distinct cortical cytoplasmic domain in **(A)**. **(D)** A lateral view of *vasa* staining shows the concave shape of the cortex at the position of the RCB and the cone-shaped morphology of the RCB itself (arrowhead). **(E)** The nucleic acid dye SYTOX Green weakly labels the RCB (arrow) and strongly labels the polar bodies (arrowhead) showing that the RCB can first be detected on the vegetal side of the embryo, opposite the polar bodies. Scale bar: **(B)** 50 μm **(C)** 25 μm **(D)** 50 μm **(E)** 100 μm. RCB, RNA-containing body.

The cortex of the one-cell *Parhyale* embryo is uniform in appearance with the exception of a cytoplasmic region that is more refringent under incident light than the surrounding cytoplasm (Figure 2A). This cytoplasmic region is first detectable as a diffuse white spot, which then forms a ring that can also be seen using incident illumination (Figure 2B) and Nomarski optics (data not shown) as a yolk-free area surrounded by a ring of small granules. We and others (M Modrell, M Gerberding and N Patel, personal communication) hypothesized that this cytoplasmic region may function as germ plasm in the specification of the germ line. Examination of the *in situ* hybridization data described above using Nomarski optics confirmed that the RCB corresponds to this refringent cytoplasmic region (Figure 2C). The localization of several germ line transcripts to this region further supports the hypothesis that it functions as germ plasm. The *in situ* hybridization staining also revealed that the cell cortex is slightly concave at the position of the RCB, and that the RCB itself is wider at the cortex and then tapers to form a rounded cone shape (Figure 2D). To our knowledge, this morphology has not been previously reported for the germ plasm of any species and its potential significance is not clear.

To investigate the characteristics of the RCB further, we stained *Parhyale* embryos with SYTOX Green, a nucleic acid dye with a high affinity for both DNA and RNA. SYTOX Green labels the polar bodies (Figure 2E, arrowhead) as well as the RCB (Figure 2E, arrow), consistent with the presence of a high concentration of germ line-associated RNAs in the RCB. *Parhyale* embryos display an obvious animal-vegetal polarity upon fertilization, with the polar bodies localized at the animal pole of the egg [35]. We observed that the RCB was generally positioned at the center of the long axis of the egg but was occasionally found asymmetrically positioned along this axis. However, it was always observed on the vegetal side of the egg, opposite the polar bodies, which mark the animal pole (Figure 2E).

We then analyzed the movement of the RCB using live imaging with incident illumination and found that just prior to the first mitotic division, this region changes morphology as the small granules that compose the ring coalesce and the yolk-free region moves below the cortex (Additional file 1). The RCB is usually at or very close to the position of the first cleavage furrow and is often enveloped within the cleavage furrow during cytokinesis (Additional file 1). In a small number of embryos where the RCB was asymmetrically positioned along the long axis of the embryo, the RCB remained at the cortex of the embryo until the second cleavage, at which point it was enveloped by the cleavage furrow and was no longer visible (Additional file 2). Asymmetric segregation of the RCB to the g blastomere would be consistent with a germ plasm function. However, due to its subcortical position after the first division, we were not able to follow it past the first cleavage using light microscopy.

### Removal of the RNA-containing body results in loss of g blastomere identity

**Figure 3:**
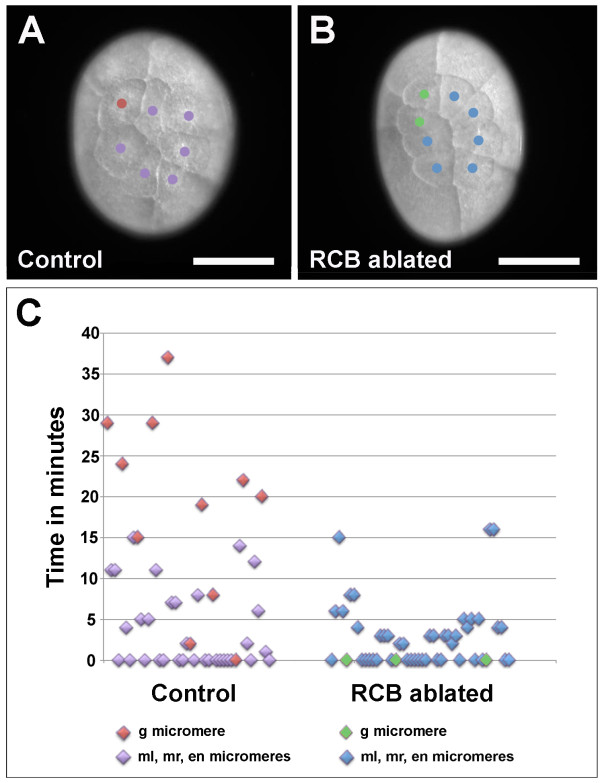
**The cell division delay that is stereotypical of the g blastomere is lost in RCB-ablated embryos. (A-B)** Still images from time-lapse recordings just subsequent to the fourth mitotic division. **(A)** Control embryo in which cytoplasm was removed from a spot approximately 100 μm from the RCB. Division of the g blastomere (red) is delayed with respect to the other micromeres (purple), which have already divided. **(B)** RCB-ablated embryo in which the g micromere (green) divided with the other micromeres (blue). **(C)** Scatter plot showing time points of the fourth mitotic division for individual micromeres. For control embryos, the g micromere is marked in red and the ml, mr and en micromeres are labeled with purple. For RCB-ablated embryos, micromeres with morphology consistent with that of g are marked with green and the ml, mr and en micromeres are labeled with blue. For RCB-ablated embryos in which g was not unambiguously identifiable based on morphology, all micromeres are marked in blue. In control embryos, division of g is generally delayed with respect to the other micromeres, while in RCB-ablated embryos, this delay in division is not observed. Scale bar: 200 μm **(A and B)** en, endoderm; g, germ line; ml, left trunk mesoderm; mr, right trunk mesoderm; RCB, RNA-containing body.

We hypothesized that if the RCB functions as germ plasm, removing it should result in a loss of germ line specification. To test this hypothesis, we physically removed it at the one-cell stage by poking a small hole in the embryo at the center of the RCB and then gently squeezing the cytoplasm out. Control embryos were pierced at a spot about 100 μm from the RCB along the long axis of the embryo. Similar amounts of cytoplasm were removed from all embryos. In the eight-cell embryo, the g micromere is specified as the exclusive source of the germ line, and it can be identified by the following morphological characteristics: it is the smallest micromere; it is the sister cell of the smallest macromere (Mav); and it does not contact the opposing micromere (en) [20]. We found that when we removed the RCB, the morphology of the blastomeres was altered so that identification of g was ambiguous, while control embryos appeared wild-type and the g blastomere could be easily identified.

g can also be identified based on the timing of its division. In wild-type embryos, the first three divisions that result in the eight-cell embryo are synchronous, while the fourth division is asynchronous, with g dividing after the other seven blastomeres [36]. We found that in 82% of control embryos (*n* = 11), g divided 8 to 37 minutes after the other micromeres (Figure 3A, red data points in C; Additional file 3). This is comparable to wild-type embryos, where it was reported that cleavage of g was delayed 10 to 60 minutes in 88% of embryos [36]. These data indicate that removal of cytoplasm from a random position close to the RCB does not affect micromere morphology or the timing of micromere divisions. We then examined embryos in which the RCB had been removed, and we found that the timing of cell division was altered so that in 100% of embryos (*n* = 12), all of the micromeres divided within 16 minutes of one another (Figure 3B,C; Additional file 3). In 75% of these embryos, the micromeres all divided within 5 minutes of one another, essentially dividing synchronously. In 3 of the 12 RCB-ablated embryos, the morphology of one of the blastomeres at the eight-cell stage was consistent with that of a g micromere. However, these micromeres did not divide later than the other micromeres (Figure 3C, green data points).

**Figure 4:**
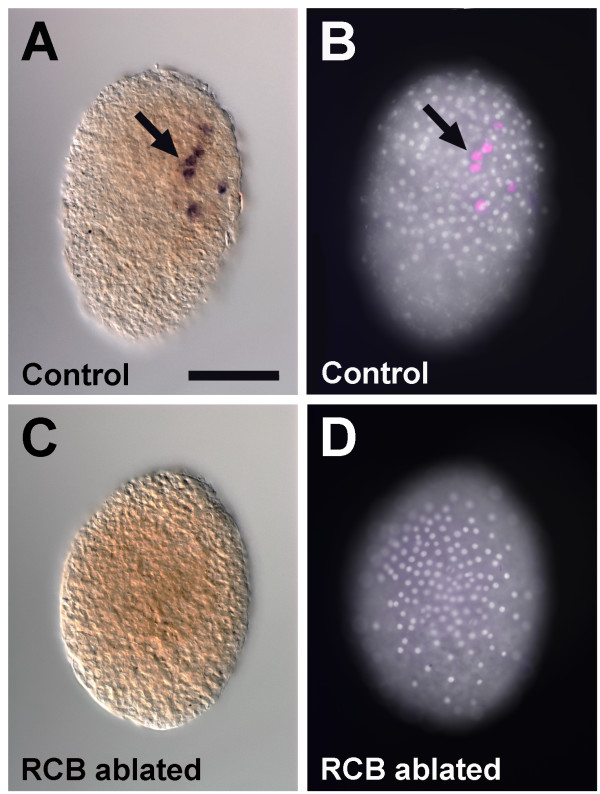
***vasa***-**positive cells are absent in RCB-ablated embryos. (A,B)** Wild-type control embryos. **(A)***vasa* is expressed in primordial germ cells in a wild-type embryo at the early germ disc stage (arrow). **(B)** False-colored *vasa in situ* hybridization (pink) with Hoechst to label the nuclei (white). **(C,D)** RCB-ablated embryos. **(C)***vasa* expression is not seen in a germ disc stage embryo, although Hoechst staining **(D)** shows that a germ disc has formed. Scale bar: 200 μm (all panels). RCB, RNA-containing body.

To determine directly whether germ cells are specified in RCB-ablated embryos, we used the germ line marker *vasa* to examine germ disc stage embryos for the presence of primordial germ cells [21,25]. In 100% of wild-type embryos, four to nine *vasa*-positive cells were present 20 to 24 hours after egg-laying (*n* = 25; Figure 4A,B). In contrast, when the RCB was removed, 97% of embryos had no *vasa*-positive cells (*n* = 32/33; Figure 4C,D). Taken together, these data support the hypothesis that the RCB is required for specification of the germ line, as the g micromere and its descendants cannot be unambiguously identified in RCB-ablated embryos based on characteristic morphology, timing of division or *vasa* expression.

### The RNA-containing body is required for correct somatic cell behavior and establishment of anterior-posterior polarity

**Figure 5:**
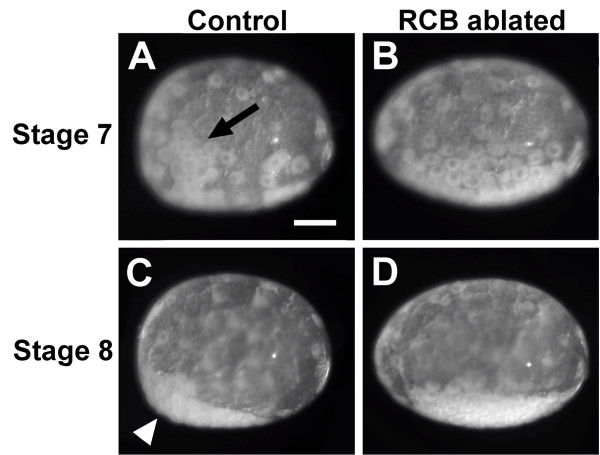
**RCB-ablated embryos do not form a rosette and display a defect in anterior-posterior polarity. (A–D)** Still images from time-lapse movies of embryos developing from eight cells to early germ disc. **(A)** A control embryo in which cytoplasm was removed from a spot approximately 100 μm from the RCB. At stage 7, descendants of the Mav macromere and ml, mr and g micromeres form a rosette structure (arrow). **(B)** In stage 7 RCB-ablated embryos, a rosette structure does not form. **(C)** During stage 8, control embryos form a germ disc at the anterior end of the embryo (arrowhead). **(D)** Stage 8 RCB-ablated embryos form a germ disc, but it does not move to the anterior of the embryo; instead the germ disc remains at the center. Scale bar: 100 μm (all panels). RCB, RNA-containing body.

Previous studies demonstrated that when the g blastomere is ablated at the eight-cell stage, the embryo develops normally but lacks germ cells [21, M Modrell, M Gerberding and N Patel, personal communication]. Surprisingly, we found that when the RCB is removed, 100% of embryos were unable to undergo gastrulation (*n* > 50). In wild-type embryos, the first cells to undergo gastrulation are descendants of g and its sister macromere Mav [36,37]. Prior to gastrulation, these cells form a cluster called a rosette, which has a stereotyped clonal composition and can be identified by its distinct morphology (Figure 5A, arrow; Additional file 4). We examined RCB-ablated embryos and found that the rosette structure does not form (*n* = 42; Figure 5B; Additional file 4), in contrast to control embryos, which undergo normal rosette formation and gastrulation (*n* = 10; Figure 5A; Additional file 4). The failure of the rosette to form in RCB-ablated embryos cannot be due to the absence of g descendant cells, since it has been demonstrated that when g is ablated at the eight-cell stage, a rosette consisting of Mav, ml and mr descendant cells still forms and the embryo undergoes normal gastrulation movements [36]. Following rosette formation, the cells of control embryos condense to form a germ disc at the anterior pole of the embryo (Figure 5C, arrowhead). In RCB-ablated embryos, a germ disc forms, but it remains in the middle of the embryo rather than moving to the anterior end (Figure 5D). Formation of a germ disc indicates that some cell movements still occur normally in RCB-ablated embryos; however, the behavior of the somatic Mav cells, which do not form a rosette or ingress, is affected.

## Discussion

Studies of germ line development in the model organisms *Drosophila*, *Caenorhabditis elegans*, *Xenopus* and zebrafish have demonstrated that a range of phylogenetically distant metazoan species utilize inheritance of maternally supplied cytoplasmic determinants to specify germ cells. Analyses of germ plasm formation in these organisms have provided us with a fundamental knowledge of this mode of germ cell specification [1,6,8,29]. Similarities in the molecular composition of germ plasm between these species are striking given the diversity of their developmental mechanisms and the hypothesized independent evolution of this mode of specification [1]. There are essential differences as well, however, including the molecular mechanisms of germ plasm nucleation and assembly, which are still not well understood even in model systems [8]. To understand the evolution of germ plasm, a comparative approach using a broader phylogenetic representation of organisms will be valuable. To this end, we investigated germ line specification in the emerging model crustacean *Parhyale hawaiensis*.

### Characterization of a putative germ plasm in *Parhyale*

In this study, we identify a distinct cytoplasmic region in the one-cell stage embryo that we term the RNA-containing body (RCB) and describe characteristics and functions of the RCB that are consistent with that of germ plasm. We found that the RCB can first be detected at the vegetal cortex of the embryo, which is where the germ line and mesendodermal lineages arise [35]. Unlike in *Drosophila*, where the posteriorly localized germ plasm is assembled during oogenesis, the RCB in *Parhyale* can first be detected following fertilization. The *Parhyale* embryo is transcriptionally quiescent until at least the 32-cell stage [36,38]; therefore, the process of germ plasm assembly must be maternally regulated, consistent with that of other model organisms. Although the mechanism by which RCB assembly occurs is not known, the invariant position of the RCB on the vegetal side of the embryo suggests that the egg may be polarized before fertilization, and an unidentified nucleator of the RCB may be localized to the future vegetal cortex during oogenesis. Alternatively, embryonic polarity may be determined by the point of sperm entry as in *C. elegans*, an organism in which the germ plasm assembles at the posterior of the embryo after fertilization [39,40]. Since we currently do not have information on the mechanism of sperm entry in *Parhyale*, it is not possible to distinguish between these alternatives.

The RCB can be identified by light microscopy as a yolk-free cytoplasmic region at the cortex of the egg. Using incident illumination and live imaging, we followed the movement of the RCB during the first embryonic cleavages. We found that the RCB is usually at or very close to the position of the first cleavage furrow and is often enveloped within the cleavage furrow during cytokinesis. In a small number of embryos where the RCB was asymmetrically positioned along the long axis of the embryo, the RCB remained at the cortex of the embryo until the second cleavage, at which point it was enveloped by the cleavage furrow and was no longer visible. Our data suggest that the RCB functions as a germ cell determinant, and therefore, we presume that it is inherited by the g blastomere, although we were not able to confirm this by light microscopy. A marker that allows live imaging of the RCB by confocal microscopy may prove useful in confirming the position of the RCB in the eight-cell embryo.

A structure similar to the RCB has not, to our knowledge, been described for other amphipod crustaceans. However, the RCB has striking similarities to a structure found in multiple penaeid shrimp species called the intracellular body (ICB) [19,41-43]. The ICB is an RNA-rich structure that was first identified in *Penaeus monodon* and hypothesized to function as a putative germ granule [41]. In penaeid shrimp, a single ICB is detectable in the D blastomere of four-cell embryos and segregates to one of two mesendoderm cells [19,41]. The descendants of one of these mesendoderm cells is hypothesized to give rise to the germ line [44-47]. Like the RCB, the ICB localizes to the cell cortex. Interestingly, an indentation of the plasma membrane adjacent to the ICB has been observed in *Marsupenaeus japonicus* embryos [43], which may be similar to the concave membrane morphology observed at the position of the RCB in *Parhyale*. Future comparative analyses will be useful in investigating the origin and function of these structures in Crustacea.

### Localization of germ line-associated RNAs to the RNA-containing body

Perhaps the best characterized aspect of germ plasm is the presence of maternally supplied RNA transcripts that are essential for germ cell formation [48]. The gene that has been most widely used to identify germ plasm as well as primordial germ cells in multiple organisms is the DEAD-box RNA helicase *vasa*[1,8,9]. Although not previously reported in a study of *Parhyale vasa* expression [25], we found that *vasa* transcripts are localized to the RCB in *Parhyale*, consistent with the localization of *vasa* to the germ plasm in other organisms. The function of *Parhyale vasa* was previously investigated using translation-blocking morpholino knock-downs, and it was found that *vasa* is required for the proliferation and maintenance of germ cells but not for their establishment [25]. These authors were not able to detect Vasa protein in germ cells until the onset of gastrulation, consistent with the morpholino phenotype. A previous study, however, reported the presence of low levels of Vasa protein in the g micromere of the eight-cell embryo, suggesting a possible function for *vasa* in specification of the germ line lineage [21]. The discrepancy between these two studies with respect to the timing of the first Vasa protein expression in germ cells may be due to the fact that these studies used two different cross-reactive anti-Vasa antibodies, raised against Vasa epitopes from a fish and an insect, respectively. To determine whether or not *vasa* functions to specify the germ line earlier in development, it will be necessary to eliminate *vasa* transcripts prior to or at the eight-cell stage. The knock-down of a maternal transcript as early as the eight-cell stage was recently reported using an siRNA [38]. Therefore, it may now be possible to investigate an earlier function for *vasa* using this reagent.

The presence of three germ line-associated transcripts (*vasa*, *orb* and *gcl*) in the RCB is consistent with a germ plasm function for this cytoplasmic region. However, if the RCB is asymmetrically segregated to the germ line during the first three cleavages, we might also expect these transcripts to localize to the ancestor cells of the g blastomere at the two- and four-cell stages and then to be enriched in g at the eight-cell stage. We suggest two possible explanations for why we do not observe this: (1) The transcripts that we examined in the RCB may be translated and subsequently degraded at the one-cell stage, and the proteins rather than the transcripts inherited by the g blastomere. (2) The transcripts may be present in the RCB at the two-, four- and eight-cell stages but are undetectable in the RCB by *in situ* hybridization due to their altered levels or intracellular localization. High concentrations of all three transcripts are found surrounding the nucleus of all blastomeres during early cleavage stages. It is thus possible that after the two-cell stage, when the RCB is no longer at the cortex, the RCB-associated transcripts become indistinguishable from those surrounding the nucleus.

### Functional analysis of the RCB

We hypothesized that the RCB is a specialized cytoplasmic region that is required for germ cell specification in *Parhyale*. To test the function of the RCB, we manually removed this cytoplasmic region and examined embryos for the presence of primordial germ cells by assaying for *vasa* expression, which is restricted to descendants of the g blastomere beginning at the 64-cell stage [25]. As predicted, we were unable to detect *vasa*-expressing cells in RCB-ablated embryos, demonstrating that the RCB is required for the specification of germ cells. In addition, we observed that in RCB-ablated embryos, the g blastomere loses its characteristic morphology as well as the cell cycle delay that follows its specification at the eight-cell stage [36]. This observation further supports our finding that the RCB is required for determination of the germ cells. The functional definition of germ plasm is that it is a localized cytoplasmic content that is both necessary and sufficient for specification of the germ line. Here we have shown that the RCB is necessary for germ line specification. Based on the loss-of-function experiments presented here, we conclude that we have identified a putative germ plasm in *Parhyale*. To test the sufficiency of the RCB in specifying the germ line, future experiments will require transplanting this cytoplasmic region to ectopic locations in the one-cell embryo or to early cleavage stage somatic blastomeres, and then examining the developing embryos for induction of ectopic germ cells.

In addition to a loss of germ cells, we found that removing the RCB affected somatic cell function. Specifically, live imaging revealed that embryos did not form a rosette, the cluster of cells composed of Mav, ml, mr and g blastomere descendants that is formed just prior to gastrulation. The rosette cells are the first to ingress during gastrulation, and we found that gastrulation did not occur in RCB-ablated embryos. This result was surprising since it had previously been shown that when the g micromere is ablated, a rosette composed of Mav, ml and mr descendants still forms and gastrulation proceeds normally [36]. This indicates that it is not the RCB ablation-induced loss of germ cell fate that causes rosette formation or gastrulation to fail. It is possible that descendants of the g micromere, which are still physically present but whose lineage is altered by ablation of the RCB, interfere with the formation of the rosette. Alternatively, it is possible that the RCB has a general function in cell fate specification and that determination of all germ layer lineages is lost. We believe that this is unlikely since the micromere descendants still migrate ventrally and the cells coalesce to form a germ disc. Rather, we suggest that the RCB is required not only for germ line specification, but may also be independently required to specify additional somatic cell fates, possibly at the first and/or second cleavages to specify the sublineages in the two- or four-cell embryo from which g will arise.

Additional roles for germ plasm in patterning animal embryos are not unusual. For example, in *Drosophila*, seven of the nine 'posterior group’ genes, which are required for anterior-posterior patterning, are also required for pole cell formation [49]. Interestingly, we also noted that while a germ disc forms in RCB-ablated embryos, it does not become localized to the anterior of the embryo as in control embryos. This phenotype may be secondary to a loss of the rosette, which in wild-type embryos marks the anterior end of the future anterior-posterior axis of the embryo [35], or it may represent an additional function for the RCB similar to the function of germ plasm in *Drosophila* anterior-posterior polarity. To further characterize the function of the RCB, it will be necessary to identify additional molecular markers of somatic cell lineages and to perform lineage analyses of these cells in RCB-ablated embryos.

## Conclusions

The data presented here describe a distinct cytoplasmic region at the cortex of the one-cell *Parhyale hawaiensis* embryo that we have termed the RNA-containing body (RCB). We show that the RCB can be identified by its morphology and by the presence of germ line-associated RNAs. Ablation of the RCB results in a loss of embryonic germ cells and a failure to gastrulate, due to aberrant somatic cell behaviors. These data support the hypothesis that the RCB has a putative germ plasm function with additional roles in somatic cell fate specification. This work provides the first functional analysis of a putative germ plasm in a crustacean and will be important for future comparative studies of germ line determination.

## Abbreviations

bp: base pair; DIG: Digoxygenin; EDTA: Ethylenediaminetetraacetic acid; El: Left anterior ectoderm; en: endoderm; Ep: Posterior ectoderm; Er: Right anterior ectoderm; g: germ line; ICB: Intracellular body; Mav: Anterior and visceral mesoderm; ml: Left trunk mesoderm; mr: Right trunk mesoderm; PBS: Phosphate-buffered saline; RCB: RNA-containing body; RT-PCR: Real-time polymerase chain reaction; SDS: Sodium dodecyl sulfate; SSC: Saline sodium citrate; siRNA: small interfering RNA.

## Competing interests

The authors declare that they have no competing interests.

## Authors’ contributions

TG proposed the idea for the research, designed and performed the experiments, analyzed the data, and wrote the manuscript. CGE analyzed the data, revised the manuscript, and obtained funding for the research. Both authors read and approved the final manuscript.

## Supplementary Material

Additional file 1 **Movement of the RCB just prior to and during the first mitotic division.** The RCB can first be detected at the cortex of the embryo and has a ring-like appearance with a yolk-free center surrounded by small granules. Just prior to cytokinesis, the RCB appears to move below the cortex and is ultimately enveloped by the cleavage furrow, thereby preventing further tracking of its movement under this light regime. Click here for file

Additional file 2 **Movement of the RCB during development from the one- to eight-cell stage.** In a small number of embryos, the RCB is asymmetrically positioned along the long axis of the embryo and remains positioned near the cortex following the first mitotic division. During the second division, the RCB is enveloped by the cleavage furrow and can no longer be tracked through subsequent divisions. Click here for file

Additional file 3 **Time-lapse movies of control and RCB-ablated embryos over a 1.25-hour period at 25°C showing the fourth mitotic division. (A)** Vegetal view of a control embryo in which cytoplasm was removed from a random position along the long axis of the embryo approximately 100 μm from the RCB. The fourth mitotic division is asynchronous and the g micromere divides after the other three micromeres. **(B)** Vegetal view of a RCB-ablated embryo in which the fourth mitotic division is synchronous and all of the blastomeres divide simultaneously. Click here for file

Additional file 4 **Time-lapse movies showing development from the eight-cell to germ disc stage. (A)** Lateral view of a control embryo. Control embryo development is identical to that of wild-type embryos with formation of a rosette structure (arrow) during stage seven and a germ disc at stage eight. The germ disc can be seen at the anterior of the embryo (anterior to the left). **(B)** Lateral view of an RCB-ablated embryo. RCB-ablated embryos do not form a rosette. A germ disc forms, but it does not move to the anterior of the embryo. Click here for file

## References

[B1] Extavour CG Akam M Mechanisms of germ cell specification across the metazoans: epigenesis and preformation Development 2003 4 24 5869 5884 10.1242/dev.00804 14597570

[B2] Mahowald AP Hennen S Ultrastructure of the 'germ plasm’ in eggs and embryos of Rana pipiens Dev Biol 1971 4 1 37 53 10.1016/0012-1606(71)90045-5 4107682

[B3] Eddy EM Germ plasm and the differentiation of the germ cell line Int Rev Cytol 1975 4 229 280 770367 10.1016/s0074-7696(08)60070-4

[B4] Nieuwkoop PD Sutasurya LA Primordial Germ Cells in the Chordates 1979 Cambridge: Cambridge University Press

[B5] Nieuwkoop PD Sutasurya LA Primordial Germ Cells in the Invertebrates: From Epigenesis to Preformation 1981 Cambridge: Cambridge University Press

[B6] Houston DW King ML Germ plasm and molecular determinants of germ cell fate Curr Top Dev Biol 2000 4 155 181 10948454 10.1016/s0070-2153(00)50008-8

[B7] Voronina E Seydoux G Sassone-Corsi P Nagamori I RNA granules in germ cells Cold Spring Harb Perspect Biol 2011 4 12 doi: 10.1101/cshperspect.a002774 10.1101/cshperspect.a002774 PMC3225947 21768607

[B8] Ewen-Campen B Schwager EE Extavour CG The molecular machinery of germ line specification Mol Reprod Dev 2010 4 1 3 18 19790240 10.1002/mrd.21091

[B9] Gustafson EA Wessel GM Vasa genes: emerging roles in the germ line and in multipotent cells Bioessays 2010 4 7 626 637 10.1002/bies.201000001 20586054 PMC3090673

[B10] Hegner RW Effects of removing the germ-cell determinants from the eggs of some chrysomelid beetles. Preliminary report Biol Bull 1908 4 19 26 10.2307/1536121

[B11] Hegner RW Experiments with chrysomelid beetles. III. The effects of killing parts of the eggs of *Leptinotarsa decemlineata* Biol Bull 1911 4 237 251 10.2307/1536101

[B12] Illmensee K Mahowald AP Transplantation of posterior polar plasm in *Drosophila*. Induction of germ cells at the anterior pole of the egg Proc Natl Acad Sci USA 1974 4 4 1016 1020 10.1073/pnas.71.4.1016 4208545 PMC388152

[B13] Tada H Mochii M Orii H Watanabe K Ectopic formation of primordial germ cells by transplantation of the germ plasm: direct evidence for germ cell determinant in *Xenopus* Dev Biol 2012 4 1 86 93 10.1016/j.ydbio.2012.08.014 23046626

[B14] Ephrussi A Lehmann R Induction of germ cell formation by oskar Nature 1992 4 6385 387 392 10.1038/358387a0 1641021

[B15] Ewen-Campen B Srouji JR Schwager EE Extavour CG Oskar predates the evolution of germ plasm in insects Curr Biol 2012 4 23 2278 2283 10.1016/j.cub.2012.10.019 23122849

[B16] Lynch JA Ozuak O Khila A Abouheif E Desplan C Roth S The phylogenetic origin of oskar coincided with the origin of maternally provisioned germ plasm and pole cells at the base of the Holometabola PLoS Genet 2011 4 4 e1002029 10.1371/journal.pgen.1002029 21552321 PMC3084197

[B17] Heming BS Origin and fate of germ cells in male and female embryos of *Haplothrips verbasci* (Osborn) (Insecta, Thysanoptera, Phlaeothripidae) J Morphol 1979 4 323 344 10.1002/jmor.1051600305 30200696

[B18] Sagawa K Yamagata H Shiga Y Exploring embryonic germ line development in the water flea, *Daphnia magna*, by zinc-finger-containing VASA as a marker Gene Expr Patterns 2005 4 5 669 678 10.1016/j.modgep.2005.02.007 15939379

[B19] Pawlak JB Sellars MJ Wood A Hertzler PL Cleavage and gastrulation in the Kuruma shrimp *Penaeus* (*Marsupenaeus*) *japonicus* (Bate): a revised cell lineage and identification of a presumptive germ cell marker Dev Growth Differ 2010 4 8 677 692 10.1111/j.1440-169X.2010.01205.x 20874712

[B20] Gerberding M Browne WE Patel NH Cell lineage analysis of the amphipod crustacean *Parhyale hawaiensis* reveals an early restriction of cell fates Development 2002 4 24 5789 5801 10.1242/dev.00155 12421717

[B21] Extavour CG The fate of isolated blastomeres with respect to germ cell formation in the amphipod crustacean *Parhyale hawaiensis* Dev Biol 2005 4 2 387 402 10.1016/j.ydbio.2004.09.030 15617682

[B22] Price AL Modrell MS Hannibal RL Patel NH Mesoderm and ectoderm lineages in the crustacean *Parhyale hawaiensis* display intra-germ layer compensation Dev Biol 2010 4 1 256 266 10.1016/j.ydbio.2009.12.006 20005872

[B23] Alwes F Hinchen B Extavour CG Patterns of cell lineage, movement, and migration from germ layer specification to gastrulation in the amphipod crustacean *Parhyale hawaiensis* Dev Biol 2011 4 1 110 123 21827744 10.1016/j.ydbio.2011.07.029

[B24] Chaw RC Patel NH Independent migration of cell populations in the early gastrulation of the amphipod crustacean *Parhyale hawaiensis* Dev Biol 2012 4 1 94 109 10.1016/j.ydbio.2012.08.012 23046627

[B25] Ozhan-Kizil G Havemann J Gerberding M Germ cells in the crustacean *Parhyale hawaiensis* depend on Vasa protein for their maintenance but not for their formation Dev Biol 2009 4 1 230 239 10.1016/j.ydbio.2008.10.028 19013453

[B26] Rehm EJ Hannibal RL Chaw RC Vargas-Vila MA Patel NH The crustacean *Parhyale hawaiensis*: a new model for arthropod development Cold Spring Harb Protoc 2009 1 pdb.emo114 doi: 10.1101/pdb.emo114. 20147009 10.1101/pdb.emo114

[B27] Rehm EJ Hannibal RL Chaw RC Vargas-Vila MA Patel NH *In situ* hybridization of labeled RNA probes to fixed *Parhyale hawaiensis* embryos Cold Spring Harb Protoc 2009 4 1 pdb.prot5130 doi: 10.1101/pdb.prot5130 10.1101/pdb.prot5130 20147025

[B28] Zeng V Extavour CG ASGARD: an open-access database of annotated transcriptomes for emerging model arthropod species Database (Oxford) 2012 4 bas048 doi: 10.1093/database/bas048. Print 2012 23180770 10.1093/database/bas048 PMC3504982

[B29] Juliano CE Swartz SZ Wessel GM A conserved germline multipotency program Development 2010 4 24 4113 4126 10.1242/dev.047969 21098563 PMC2990204

[B30] Jongens TA Hay B Jan LY Jan YN The *germ cell-less* gene product: a posteriorly localized component necessary for germ cell development in *Drosophila* Cell 1992 4 4 569 584 10.1016/0092-8674(92)90427-E 1380406

[B31] Lantz V Ambrosio L Schedl P The *Drosophila* orb gene is predicted to encode sex-specific germline RNA-binding proteins and has localized transcripts in ovaries and early embryos Development 1992 4 1 75 88 1638994 10.1242/dev.115.1.75

[B32] Rangan P DeGennaro M Jaime-Bustamante K Coux RX Martinho RG Lehmann R Temporal and spatial control of germ-plasm RNAs Curr Biol 2009 4 1 72 77 10.1016/j.cub.2008.11.066 19110432 PMC2766415

[B33] Shippy TD Yeager SJ Denell RE The *Tribolium spineless* ortholog specifies both larval and adult antennal identity Dev Genes Evol 2009 4 1 45 51 10.1007/s00427-008-0261-9 19030877 PMC2605184

[B34] Rogers BT Peterson MD Kaufman TC The development and evolution of insect mouthparts as revealed by the expression patterns of gnathocephalic genes Evol Dev 2002 4 2 96 110 10.1046/j.1525-142X.2002.01065.x 12004967

[B35] Browne WE Price AL Gerberding M Patel NH Stages of embryonic development in the amphipod crustacean, *Parhyale hawaiensis* Genesis 2005 4 3 124 149 10.1002/gene.20145 15986449

[B36] Alwes F Hinchen B Extavour CG Patterns of cell lineage, movement, and migration from germ layer specification to gastrulation in the amphipod crustacean *Parhyale hawaiensis* Dev Biol 2011 4 1 110 123 10.1016/j.ydbio.2011.07.029 21827744

[B37] Price AL Patel NH Investigating divergent mechanisms of mesoderm development in arthropods: the expression of Ph-twist and Ph-mef2 in *Parhyale hawaiensis* J Exp Zool B Mol Dev Evol 2008 4 1 24 40 17152085 10.1002/jez.b.21135

[B38] Nestorov P Battke F Levesque MP Gerberding M The maternal transcriptome of the crustacean *Parhyale hawaiensis* is inherited asymmetrically to invariant cell lineages of the ectoderm and mesoderm PLoS One 2013 4 2 e56049 10.1371/journal.pone.0056049 23418507 PMC3572164

[B39] Strome S Wood WB Generation of asymmetry and segregation of germ-line granules in early C. elegans embryos Cell 1983 4 1 15 25 10.1016/0092-8674(83)90203-9 6684994

[B40] Schneider SQ Bowerman B Cell polarity and the cytoskeleton in the *Caenorhabditis elegans* zygote Annu Rev Genet 2003 4 221 249 10.1146/annurev.genet.37.110801.142443 14616061

[B41] Biffis C Alwes F Scholtz G Cleavage and gastrulation of the dendrobranchiate shrimp *Penaeus monodon* (Crustacea, Malacostraca, Decapoda) Arthropod Struct Dev 2009 4 6 527 540 10.1016/j.asd.2009.06.003 19573622

[B42] Foote A Sellars M Coman G Merritt D Cytological defects during embryogenesis in heat-induced tetraploid Kuruma shrimp *Penaeus japonicus* Arthropod Struct Dev 2010 4 4 268 275 10.1016/j.asd.2009.12.001 20060492

[B43] Grattan RM McCulloch RJ Sellars MJ Hertzler PL Ultrastructure of putative germ granules in the penaeid shrimp *Marsupenaeus japonicus* Arthropod Struct Dev 2013 4 2 153 164 10.1016/j.asd.2012.11.002 23183128

[B44] Zilch R Embryologische Untersuchungen an der holoblastischen Ontogenese vonPenaeus trisulcatus Leach (Crustacea, Decapoda) Zoomophologie 1978 4 67 100 10.1007/BF00993744

[B45] Zilch R Cell lineage in arthropods? Fortschritte in der zoologischen Systematik und Evolutionsforschung 1979 4 19 41

[B46] Hertzler PL Development of the mesendoderm in the dendrobranchiate shrimp *Sicyonia ingentis* Arthropod Struct Dev 2002 4 1 33 49 10.1016/S1467-8039(02)00018-X 18088969

[B47] Hertzler PL Cleavage and gastrulation in the shrimp *Penaeus* (*Litopenaeus*) *vannemei* (Malacostraca, Decapoda, Dendrobranchiata) Arthropod Struct Dev 2005 4 455 469 10.1016/j.asd.2005.01.009 19162223

[B48] Zhou Y King ML Sending RNAs into the future: RNA localization and germ cell fate IUBMB Life 2004 4 1 19 27 10.1080/15216540310001658886 14992376

[B49] Manseau LJ Schupbach T The egg came first, of course! Anterior-posterior pattern formation in *Drosophila* embryogenesis and oogenesis Trends Genet 1989 4 12 400 405 2696183 10.1016/0168-9525(89)90198-4

